# PYED-1 Inhibits Biofilm Formation and Disrupts the Preformed Biofilm of *Staphylococcus aureus*

**DOI:** 10.3390/antibiotics9050240

**Published:** 2020-05-08

**Authors:** Adriana Vollaro, Anna Esposito, Eliana Pia Esposito, Raffaele Zarrilli, Annalisa Guaragna, Eliana De Gregorio

**Affiliations:** 1 Department of Molecular Medicine and Medical Biotechnology, University of Naples Federico II, 80131 Naples, Italy; vollaroadriana@libero.it; 2 Department of Chemical Sciences, University of Naples Federico II, 80126 Naples, Italy; anna.esposito5@unina.it; 3 Department of Public Health, University of Naples Federico II, 80131 Naples, Italy; elianapia.esposito@unina.it (E.P.E.); rafzarri@unina.it (R.Z.)

**Keywords:** biofilm formation, preformed biofilm, antibiofilm activity, biofilm eradication agent, *Staphylococcus aureus*, corticosteroid

## Abstract

Pregnadiene-11-hydroxy-16α,17α-epoxy-3,20-dione-1 (PYED-1), a heterocyclic corticosteroid derivative of deflazacort, exhibits broad-spectrum antibacterial activity against Gram-negative and Gram-positive bacteria. Here, we investigated the effect of PYED-1 on the biofilms of *Staphylococcus aureus*, an etiological agent of biofilm-based chronic infections such as osteomyelitis, indwelling medical device infections, periodontitis, chronic wound infections, and endocarditis. PYED-1 caused a strong reduction in biofilm formation in a concentration dependent manner. Furthermore, it was also able to completely remove the preformed biofilm. Transcriptional analysis performed on the established biofilm revealed that PYED-1 downregulates the expression of genes related to quorum sensing (*agrA*, *RNAIII*, *hld*, *psm*, and *sarA*), surface proteins (*clfB* and *fnbB*), secreted toxins (*hla*, *hlb,* and *lukD*), and capsular polysaccharides (*capC*). The expression of genes that encode two main global regulators, *sigB* and *saeR*, was also significantly inhibited after treatment with PYED-1. In conclusion, PYED-1 not only effectively inhibited biofilm formation, but also eradicated preformed biofilms of *S. aureus*, modulating the expression of genes related to quorum sensing, surface and secreted proteins, and capsular polysaccharides. These results indicated that PYED-1 may have great potential as an effective antibiofilm agent to prevent *S. aureus* biofilm-associated infections.

## 1. Introduction

One of the bacterial growth modes is the development of biofilms, which may be considered a basic survival strategy in hostile environments [1]. Biofilms are sessile communities of bacterial cells, attached to each other and/or to surfaces, embedded in a self-produced matrix of extracellular polymeric substances (EPS) [2]. Biofilm formation plays a crucial role in bacterial infection and antimicrobial resistance, because biofilm-embedded bacteria are more resistant to common antimicrobial agents and host defense systems than bacteria in the planktonic state [3]. Increasing evidence demonstrates that cells in biofilms on a biotic or abiotic surface are 1000-fold more resistant to conventional drugs than planktonic cells [4,5]. Once established, biofilms become difficult to eradicate, leading to chronic and persistent infections [6]. *Staphylococcus aureus* is the major Gram-positive pathogen which causes biofilm-associated infections, because of its ability to form biofilms on a wide range of surfaces [7,8].

As a result of this, there is an urgent need to develop new agents that inhibit *S. aureus* biofilm formation, and/or disrupt established biofilms [9]. In previous studies, we showed that pregnadiene-11-hydroxy-16α,17α-epoxy-3,20-dione-1 (PYED-1) exhibits effective antibacterial activity against *S. aureus* ATCC 29213 and *A. baumannii* ATCC 17978, without cytotoxic effects [10,11]. Additionally, we demonstrated that PYED-1 at sub-inhibitory concentrations hinders the biofilm formation of the *Stenotrophomonas maltophilia* K279a strain [12].

In this study, we investigated the in vitro effect of PYED-1 on biofilm formation and eradication of preformed biofilm by *S. aureus*. 

## 2. Results and Discussion

### 2.1. Effect of PYED-1 on S. aureus Biofilm Formation

*S. aureus* has the ability to produce a biofilm, which protects it from the action of antibacterial drugs [13]. The development of antibiofilm agents may be a potential approach for the management of disease progression, and elimination of this pathogen from the target site of infection [14]. Previous studies have shown that higher doses of topical corticosteroids (budesonide, mometasone, and fluticasone), commonly used in the treatment of chronic rhinosinusitis, have effective antibiofilm activity against *S. aureus* [15]. Recent studies have shown that PYED-1 at 16 μg/mL exerts effective inhibitory activity of planktonic cell growth against *S. aureus* ATCC 29213 [10,11]. 

**Figure 1:**
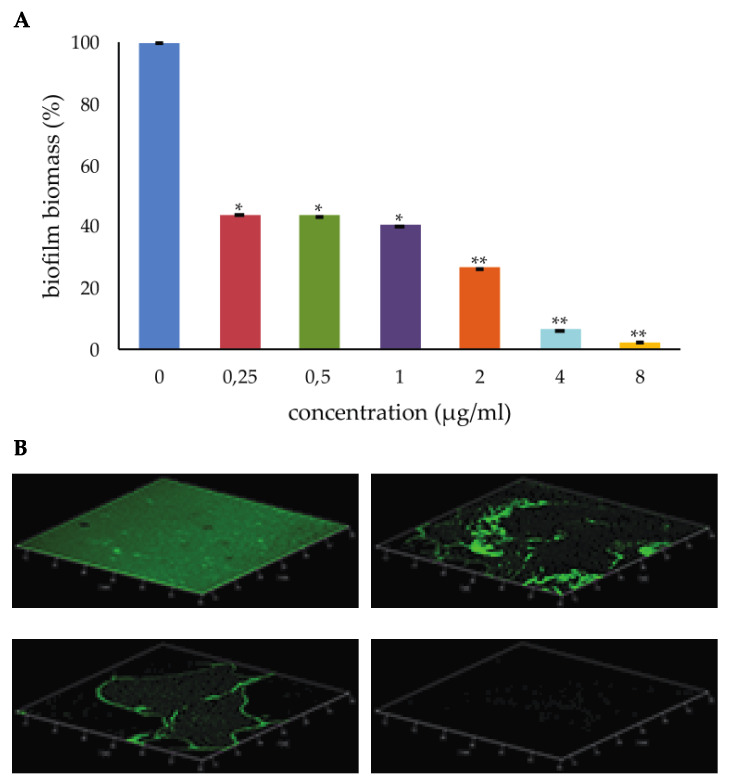
Inhibitory effect of pregnadiene-11-hydroxy-16α,17α-epoxy-3,20-dione-1 (PYED-1) on *S. aureus* biofilm formation. (**A**) Biofilms were quantified after crystal-violet staining. Values are presented as means ±SDs. Asterisks indicate statistically significant differences between the treated and untreated biofilms (*p < 0.05 and **p < 0.01, respectively). (**B**) Confocal laser scanning microscopy (CLSM) analysis of the biofilm formed by *S. aureus* ATCC 29213 in the absence (left upper panel) or presence of PYED-1, at the concentrations of 2 µg/mL (right upper panel), 4 µg/mL (left inferior panel), and 8 µg/mL (right inferior panel).

*S. aureus* cells were treated with PYED-1 at sub-minimal inhibitory concentrations (MICs) (0.25 to 8 µg/mL), and the biofilm biomass was quantified by crystal violet (CV) staining assay. PYED-1 caused a two-fold inhibition of biofilm formation at 0.25 µg/mL (1/64× MIC). When PYED-1 was added at 8 µg/mL (1/2 × MIC), complete inhibition of biofilm formation was observed (Figure 1A). 

Furthermore, PYED-1 at concentrations of up to 8 µg/mL did not impair planktonic growth at concentrations of up to 8 µg/mL (data not shown), thus suggesting that the reduction of biofilm formation caused by PYED-1 was due to its antibiofilm activity, and not to its antimicrobial activity. Similar results were obtained in previous studies on *S. maltophilia* [12], in which treatment with PYED-1 at 1/4× MIC, 1/8× MIC, and 1/16× MIC was able to inhibit biofilm formation by 97%, 90%, and 57%, respectively, compared with the untreated control. Biofilm formation was also assessed qualitatively using confocal laser scanning microscopy (CLSM). *S. aureus* cells were incubated with or without 2, 4, and 8 µg/mL of PYED-1 for 24 h and stained using SYTO 9 and propidium iodide fluorophores to visualize live and dead cells, respectively (Figure 1B). The CLSM results confirmed the inhibition of biofilm formation in a concentration-dependent manner. This analysis revealed a thick biofilm coverage in the control sample, while PYED-1 treated samples showed a visible reduction in the coverage of biofilm. A clear reduction of the number of bacteria was observed even at the concentration of 2 μg/mL PYED-1 (Figure 1B, right upper panel). Treatment of the biofilm with 8 μg/mL PYED-1 for 24 h resulted in the complete absence of adherent cells (Figure 1B, right inferior panel).

The ability of PYED-1 to completely inhibit *S. aureus* biofilm formation makes it a promising drug to control *S. aureus* biofilm growth. 

### 2.2. Effect of PYED-1 against S. aureus Preformed Biofilm

**Figure 2:**
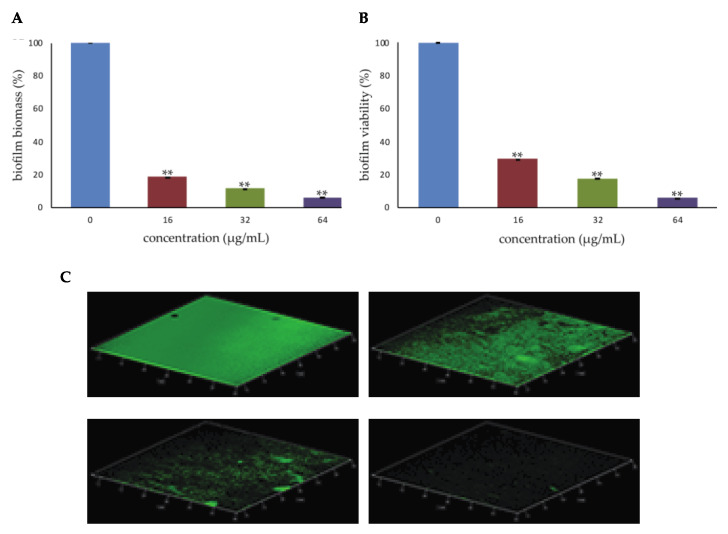
Eradicating effect of PYED-1 on *S. aureus* preformed biofilm. Biofilm formed after 48 h in 96-well microplates was treated with different PYED-1 concentrations for 24 h at 37 °C under static conditions. (**A**) Biofilm biomass were measured by crystal violet (CV) staining. (**B**) Biofilm viability was measured by tetrazolium salt reduction (XTT) assay. Error bars represent the standard deviation (SD) of three independent experiments. Asterisks indicate statistically significant differences between the treated and untreated biofilms (**p < 0.01). (**C**) CLSM analysis of preformed *S. aureus* ATCC 29213 biofilm without treatment (left upper panel) or treated with PYED-1 at 16 µg/mL (right upper panel), 32 µg/mL (left inferior panel), and 64 µg/mL (right inferior panel) for 24 h.

Mature biofilms are more difficult to treat than those at early stages, because they represent a physical barrier to drug crossing and exhibit increased drug resistance [14]. In this regard, infections in which the formation of biofilms represents a severe complication might be eradicated by using antibiofilm agents that weaken or destroy preformed biofilm cells [16], ultimately leading to biofilm disappearance [17]. As a result, that complete inhibition of biofilm formation was obtained following treatment with PYED-1 concentrations corresponding to 1/2× MIC (Figure 1), PYED-1 concentrations higher than those inhibiting biofilm growth were analyzed for their effects on preformed biofilms. To investigate this issue, one day old biofilms were exposed to PYED-1 at the concentration of 1× MIC (16 μg/mL), 2× MIC (32 μg/mL), and 4× MIC (64 μg/mL) for 24 h. Biofilm biomass and biofilm viability were measured by CV staining and tetrazolium salt reduction (XTT) assay, respectively. PYED-1 treatment at 1× MIC, 2× MIC, and 4× MIC values decreased biofilm biomass by 80%, 90%, and 94% compared to the untreated biofilm, respectively (Figure 2A). The biofilm metabolic activity, as assessed through XTT assay, showed that PYED-1 reduced the viability of *S. aureus* biofilm cells by 70%, 80% and 95% at the concentration of 16 μg/mL, 32 μg/mL, and 64 μg/mL, respectively (Figure 2B). 

Visualization of the biofilms by CLSM confirmed these results. One-day-old *S. aureus* biofilms were treated with 16, 32, or 64 µg/mL PYED-1 for 24 h, stained with the Baclight Live/Dead reagent, and observed using CLSM (Figure 2C).The CLSM results showed that increasing PYED-1 concentrations reduced the number of adherent bacteria to the slide, and consequently the biofilm biomass. Biofilm formation reduces penetration of most antibiotics, and thus their effectiveness [18]. Moreover, a slow penetration of antibiotics into bacterial cells can induce an adaptive phenotypic response that might potentially increase tolerance [19]. Furthermore, bacteria in biofilms are poorly eliminated by the immune system [18,20]. 

Consequently, the finding that PYED-1 is able to destroy existing biofilms and inhibit the continued formation of biofilms has relevance, making this substance attractive for potential use in therapeutic regimens. 

### 2.3. Biofilm Gene Expression

**Figure 3:**
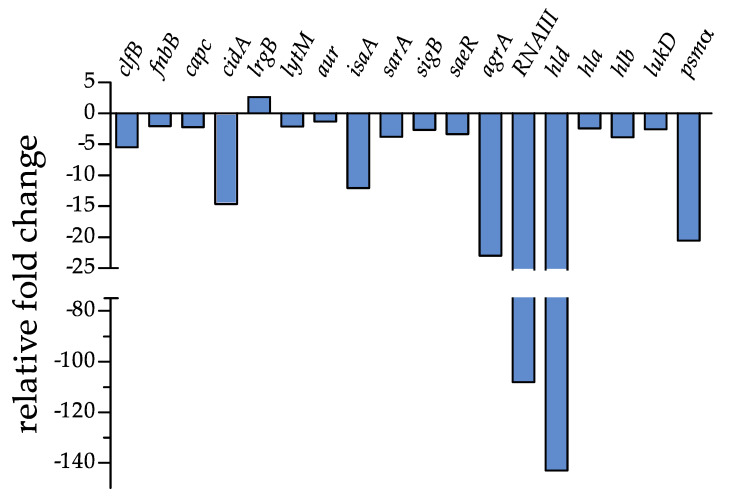
Transcriptional profile changes in *S. aureus* after treatment with PYED-1, determined by qRT-PCR with respect to *rpoB* expression. Fold-changes were calculated using treated versus untreated *S. aureus* cells. Gene descriptions, fold changes, standard deviations (SD), and *p*-values are reported in Table 1.

Our previous data showed that PYED-1 inhibition of *S. aureus* biofilm formation is associated with reduced expression of several genes involved in *S. aureus* virulence [11]. Here, we investigated the molecular mechanism responsible for the eradication activity of PYED-1. In an attempt to understand how PYED-1 removes established biofilms, we measured the transcriptional responses of *S. aureus* preformed biofilm cells exposed to a 1 x MIC value of PYED-1. RNA was purified from the samples and analyzed by quantitative RT-PCR (qRT-PCR) to assess the effect of PYED-1 exposure on the gene expression of the biofilm cells (Figure 3).

The extracellular matrix of *S. aureus* biofilm, composed of polysaccharide intracellular adhesin (PIA), teichoic acids, extracellular DNA (eDNA), and several surface proteins, is crucial for the structural integrity of biofilms [21]. Analyses of differentially expressed genes revealed that most of the analyzed genes involved in the maintenance of mature biofilms were downregulated, except the *lrgB* gene (Table 1 and Figure 3). Positively charged cytoplasmic proteins, among which fibronectin-binding proteins A and B (FnbA and FnbB) and clumping factors A and B (ClfA and ClfB), interact with eDNA released during cell autolysis and negatively charged phospholipids and teichoic acids [22]. The addition of PYED-1 to preformed *S. aureus* biofilms affected expression of both the *fnbB* and *clfB* genes (Table 1 and Figure 3).

DNA released by cell autolysis is an essential component of the *S. aureus* biofilm matrix [23]. The release of DNA is regulated by the *cid* and *lrg* genes [24,25]. The *cidA* gene encodes a murein hydrolase regulator that promotes cell lysis, while *lrg genes* encode proteins that inhibit cell lysis by preventing homo-oligomerization of *cid* gene products [26]. The transcript levels of the positive regulator *cidA* were notably decreased (about a 15-fold reduction), and the transcript levels of the negative regulator of autolysis *lrgB* were increased 2.65-fold (Table 1 and Figure 3). These results suggest that PYED-1 could reduce *S. aureus* preformed biofilms by inhibiting autolysis. Our results are in agreement with previous studies showing that licochalcone A, tea tree oil, and magnolol reduce *S. aureus* biofilm production by reducing the expression of *cidA* and increasing the expression of *lrgB* [27,28,29]. 

We also examined the expression of autolysin-encoding genes. PYED-1 significantly reduced the expression of the *lytM* gene, which encodes a glycyl-glysine endopeptidase (Table 1 and Figure 3). This is in agreement with previous studies showing that the exposure of *S. aureus* biofilm to the lytic proteins LysH5 and CHAPSH3b downregulates several autolysin-encoding genes [30]. The transcriptional levels of *isaA* (immunodominant staphylococcal antigen) were also markedly (12-fold) reduced (Table 1 and Figure 3). In contrast, the transcriptional levels of *isaA* were increased by PYED-1 in planktonic cells [11]. Changes in the transcriptional control of *isaA* in planktonic cells and preformed biofilms were also observed when treating planktonic and biofilm cells with secalonic acid D [31].

Several global regulators, such as the accessory gene regulator (*agr*) system, sigma factor B (SigB), staphylococcal accessory regulator (SarA), and two component system SaeRS, modulate biofilm formation in *S. aureus* [32]. The expression of the *saeR* and *sarA* genes was downregulated 3.34- and 3.77-fold by PYED-1 treatment, respectively (Table 1 and Figure 3). The SaeR regulator controls the expression of genes encoding major virulence factors, such as *hla* and *hlb* (α-and β-hemolysin), *coa* (coagulase), *eap* (extracellular adherence protein), and *fnbA/B* genes [33], some of which contribute to the ability of *S. aureus* to survive in neutrophils [34]. Indeed, the downregulation of the *sae* operon may reduce the escape of *S. aureus* from PMNs [35]. The SarA regulator enhances the transcription of matrix adhesion genes, including *fnbA* and *fnbB* [36]. The mutation of the *sarA* gene reduces accumulation of α-toxin and phenol soluble modulins (PSMs) [37]. Not surprisingly, the levels of some genes regulated by the two regulators, such *fnbB, hla*, *hlb,* and *psm,* were also notably reduced (Table 1 and Figure 3). PSMs [21] and beta-hemolysin [38] have been shown to bind eDNA, promoting the formation of amyloid-like fibers that stabilize the extracellular matrix and consequently *S. aureus* biofilms. A minor accumulation of PSMs was observed in a *sarA* mutant [39], therefore the observed reduction of *psm* gene expression could also be correlated with the downregulation of the *sarA* gene. Moreover, SaeR and SarA also synergistically repress the production of proteases [39], which are involved in biofilm detachment. We speculated that the downregulation of *sae*RS and *sarA* by PYED-1 treatment may also promote the production of extracellular nucleases and proteases, limiting the accumulation of the eDNA and proteins that promote biofilm formation, thus favoring biofilm detachment. In further support of this hypothesis, compounds targeting the expression of *sarA* have been shown to have potent antibiofilm and anti-virulence activity [40,41].

In the conditions under which we aimed to remove biofilms, the *agrA* gene and the small non-coding *RNAIII*, associated with the quorum sensing systems of *S. aureus,* were markedly downregulated by 22.99- and 108.14-fold, respectively, following treatment with a 1 × MIC value of PYED-1 (Table 1 and Figure 3). The transcript levels of *hld*, which encodes delta-hemolysin, were downregulated by a factor of 143.04. The *agr* expression was activated by SarA, therefore the observed reduction of *agr* expression could be also correlated with the downregulation of the *sarA* gene. In turn, RNAIII regulates the expression of secreted virulence factors, including the alfa-, beta-, and delta-hemolysins [42].

The alternative sigma factor, SigB, that leads global changes in gene expression, is known to control *sarA* expression [43]. After treatment of the preformed biofilms with PEYD-1 the expression of the *sigB* gene was downregulated 2.67-fold (Table 1 and Figure 3). A lower *sigB* expression could partly account for the *sarA* downregulation observed in our experiments. It has been shown that *sigB*-deficient *S. aureus* are unable to form biofilms and feature increased RNA III production [44], leading to up-regulation of proteases. PYED-1 decreased the RNA III level, suggesting that up-regulation of the RNAIII level via a decreased SigB level could be compensated for by changes in other genes that may also regulate the RNAIII level. 

Exposure to a 1 × MIC value of PYED-1 moderately reduced the expression of the *capC* gene (Table 1 and Figure 3). Reduced capsule production may render an organism more sensitive to phagocytosis [45]. 

Based on the above findings, we conclude that the eradication of *S. aureus* preformed biofilms is associated with downregulation of the expression of several biofilm- and toxin-related genes. 

## 3. Materials and Methods

### 3.1. Effect of PYED-1 on S. aureus Biofilm Formation

The chemical synthesis and structural characterization of PYED-1 was realized as previously reported [10,11]. PYED-1 was dissolved in dimethyl sulfoxide (DMSO) to the concentration of 50 mg/mL. Two-fold serial dilutions of the compounds were prepared in ultrapure DNase/RNase-free distilled water (Thermo Fisher Scientific, catalog number 10977035). The biofilm prevention efficacy of PYED-1 was assayed using the crystal violet (CV) biofilm staining method [46]. A bacterial cell suspension (5 × 10^6^ cells/mL in TSB supplemented with 0.5% glucose) was aliquoted (100 μL/well) in a 96-well flat bottomed polystyrene microtiter plate, treated or not with 100 μL of scalar doses of PYED-1 (the concentrations ranged from 0.25 μg/mL to 8 μg/mL). Non-treated bacteria were incubated with 100 μL of broth containing scalar doses of DMSO (range concentrations from 0.0005% to 0.016%) and used as the control. After 24 h of incubation at 37 °C, the supernatant was gently removed, and the wells were washed twice with 200 μl of phosphate-buffered saline (PBS) 1X pH 7.4 (Thermo Fisher Scientific, catalog number 10010015). The plates were dried at 60 °C for 30 min, and the biofilms were stained with 200 μL of 0.1% crystal violet for 15 minutes. After washing with 200 μL of PBS 1×, the wells were filled with 200 μL of 96% ethanol, incubated for 20 minutes at room temperature, and the absorbance was measured at 595 nm using a microplate reader (Bio-Rad Laboratories S.r.l.). The percentage of biofilm mass reduction was calculated as follows: [(Ac−At)/Ac] × 100, where Ac is the OD595 for the control well and At is the OD595 for the biofilm in the presence of PYED-1. All data points are expressed as means ± SDs of three separate experiments performed in triplicate.

### 3.2. Effect of PYED-1 against Preformed Biofilm Biomasses

Biofilms were allowed to form in each well of a 96-well microtiter plate, as described above. After 24 h the planktonic cells were gently removed by aspiration, and the plate was washed with 200 μL of PBS 1×. Two hundred microliters of PYED-1 was added at concentrations ranging from 16 μg/mL to 64 μg/mL, and the plate was incubated for 24 h at 37 °C. Non-treated cells were incubated with 200 μL of broth containing scalar doses of DMSO (concentrations ranging from 0.032% to 0.128%). Following the incubation, crystal violet-staining was performed to assess the biofilm biomass. All data points are expressed as means ± SDs of three separate experiments performed in triplicate.

### 3.3. Effect of PYED-1 against Preformed Biofilm Viability

Biofilms were allowed to form in each well and were treated following 24 h of incubation, as described above. After PYED-1 treatment, 150 µl of a mixed solution of XTT [2,3-bis(2-methyloxy-4-nitro-5-sulfophenyl)-2H-tetrazolium-5-carboxanilide] and N-methyl dibenzopyrazine methyl sulfate (Roche Diagnostics) was added to each well. Following incubation in the dark for 40 min at 37 °C, the biofilm metabolic activity was determined through the measurement of the absorbance value at 490 nm, using a microplate reader (Bio-Rad Laboratories S.r.l.). Viability values were compared with respect to control samples treated with scalar doses of DMSO (concentrations ranging from 0.032% to 0.128%). All data points are expressed as means ± SDs of three separate experiments performed in triplicate. 

### 3.4. Confocal Laser Scanning Microscopy (CLSM)

For CLSM analysis of biofilm formation, *S. aureus* cells were allowed to form biofilms in chambered cover glasses (μ Slide 4 well; Ibidi GmbH, Munich, Germany) in the presence of PYED-1 (concentrations ranged from 2 μg/mL to 8 μg/mL) or of scalar doses of DMSO (concentrations ranging from 0.032% to 0.128%) for 24 h at 37 °C in static conditions. For CLSM analysis of preformed biofilms, the biofilms were allowed to form in each chambered cover glass and then were treated with PYED-1 (concentrations ranging from 16 μg/mL to 64 μg/mL) or scalar doses of DMSO (concentrations ranging from 0.032% to 0.128%) for 24 h at 37 °C in static conditions. Then, the biofilms were washed with sterile PBS 1× and stained with LIVE/DEAD BacLight Bacteria Viability stains (Life Technologies, Monza, Italy), a mixture of green-fluorescent nucleic acid stain SYTO 9 and the red-fluorescent nucleic acid stain propidium iodide (PI). Briefly, 200 μL of dye solution was added to the well and incubated at room temperature for 15 min in the dark. After incubation, the stain was removed, and wells were washed with distilled water. Stained biofilms were observed using an LSM 700 inverted confocal laser-scanning microscope (Zeiss, Arese, Milan, Italy). Three different areas of each well were scanned using a 10× lens. Signals were recorded in the green channel for SYTO 9 (excitation 488 nm, emission 500–525 nm) and in the red channel for PI (excitation 500–550 nm, emission 610–650 nm). For CLSM analysis of the preformed biofilms, PYED-1 was added to 1-day-old biofilms at 16 μg/mL, 32 μg/mL, and 64 μg/mL. Untreated bacterial suspensions were used as the control. After 24 h, the biofilms were rinsed with PBS 1× and stained with LIVE/DEAD BacLight Bacteria Viability stains, as described above.

### 3.5. Biofilm Gene Expression

For RNA isolation, *S. aureus* ATCC 29213 was grown in 24-well polystyrene tissue culture plates containing TSB supplemented with 0.5% glucose and incubated at 37 °C. After 24 h of biofilm growth, the suspension cultures were removed from each well. The plates were washed twice with sterile PBS 1×. A total of 600 μL of fresh medium with 0.032% DMSO or PYED-1 (final concentration 16 μg/mL) was added to the wells, and the plate was incubated at 37 °C for 16 h without shaking. The plates were then washed with PBS 1× (to remove planktonic cells) and the adherent cells were scraped with a pipettor and placed in the RNAprotect Bacteria Reagent (Qiagen, Germany). The sessile cell suspension was then transferred to a microcentrifuge tube and incubated for 5 min at room temperature to stabilize the mRNA. Next, the cell suspensions were centrifuged at 5000× g for 10 min to pellet the cells, and the supernatant was decanted. Total RNA was purified according to the previously reported method [47], with some modifications. Pellets were resuspended in 200 μL of 20 mg/mL proteinase K and 200 μL TE buffer (30 mM Tris HCl, 1 mM EDTA, pH 8.0) containing 20 mg/mL lysozyme and 12.5 μg/mL lysostaphin, and incubated at 37 °C for 1 h. RNA isolation was performed using the RNeasy Mini Kit (Qiagen), according to the manufacturer’s recommended protocol. Residual DNA was removed with the DNase Max Kit (Qiagen). RNA was quantified using a Nano-drop instrument (Thermo Fisher). Total RNA was reverse transcribed into cDNA using the QuantiTect Reverse Transcription Kit (Qiagen), according to the manufacturer’s protocol. The RT-PCR was carried out in a 20 μL volume, as previously described [48], using an SYBR Green master mix (Applied Biosystems). The primer pairs used in the PCR experiments are reported in Table 2.

The expressions of genes of interest were normalized to the housekeeping gene *rpo*B. RNA samples not treated with reverse transcriptase were routinely included as no template controls. Changes in transcript levels were determined using the 2^−ΔΔCT^ method [49]. RNA expression levels were determined by using three independent cultures, and all analyses were performed in triplicate.

### 3.6. Statistical Analysis

All statistical analyses were performed with GraphPad prism 8 software (GraphPad, San Diego, CA, USA). Arithmetic means and standard deviations were used to statistically analyze continuous variables. Statistical differences between PYED-1 treated and untreated biofilms were analyzed by Student’s t test. A *p*-value < 0.05 and < 0.001 in comparison with untreated controls was considered significant.

## 4. Conclusions

The results shown herein demonstrate that PYED-1 is able to suppress the formation of *S. aureus* biofilm, as well as to disrupt *S. aureus* preformed biofilm. Our data also show that the eradication of *S. aureus* preformed biofilm is associated with downregulation of the expression of several biofilm- and toxin-related genes. Based on the above findings, we speculate that PYED-1 could be a promising biofilm inhibitor and biofilm destroyer, and a potential candidate drug for therapeutic regimens, with the aim of reducing the morbidity of *S. aureus* biofilm-related infections.

## Figures and Tables

**Table 1:** RT-PCR analysis of biofilm gene expression in *S. aureus* ATCC 29213 in the presence of PYED-1.

Gene	Description	Fold Change ± SD	*p*-Value
*clfB*	clumping factor B	−5.44 ± 0.011	0.0004
*fnbB*	fibronectin-binding protein B	−2.03 ± 0.029	0.0025
*capC*	capsule biosynthesis protein C	−2.22 ± 0.027	0.0019
*cidA*	holin-like murein hydrolase modulator	−14.67 ± 0.004	<0.0001
*lrgB*	antiholin-like protein B	+2.65 ± 0.160	0.0013
*lytM*	peptidoglycan hydrolase	−2.09 ± 0.028	0.0022
*aur*	aureolysin, zinc metalloproteinase	−1.32 ± 0.045	0.0155
*isaA*	immunodominant staphylococcal antigen	−12.06 ± 0.005	<0.0001
*sarA*	Transcriptional regulator	−3.77 ± 0.016	0.0007
*sigB*	RNA polymerase sigma factor B	−2.67 ± 0.022	0.0013
*saeR*	response regulator SaeR	−3.34 ± 0.018	0.0008
*agrA*	accessory gene regulator protein A	−22.99 ± 0.002	0.0001
*RNAIII*	small regulatory RNA	−108.14 ± 0.0005	<0.0001
*hld*	delta-haemolysin gene	−143.04 ± 0.0004	<0.0001
*hla*	alpha-haemolysin	−2.41 ± 0.025	0.0016
*hlb*	beta-haemolysin	−3.83 ± 0.015	0.0007
*lukD*	pore-forming leukocidin	−2.56 ± 0.023	0.0014
*psm* *α*	Phenol-soluble modulin	−20.54 ± 0.002	0.0001
*sarA*	Transcriptional regulator	−3.77 ± 0.016	0.0007

**Table 2:** Gene target list and oligonucleotide sequences.

Gene	Forward primer (5’-3’)	Reverse primer (5’-3’)
*agr*A	TGCGAAGACGATCCAAAAC	TTTAGCTTGCTCAAGCACCTC
*aur*	GATGGTCGCACATTCACAAG	CGCCTGACTGGTCCTTATATTC
*cap*C	CATCCAGAGCGGAATAAAGC	CGGAAATACCCGCTAATGAC
*cid*A	CTTAGCCGGCAGTATTGTTG	GTTTGCACCGTCTTCTACCC
*clf*B	TTATGGTGGTGGAAGTGCTG	TGGACTTGGTTCTGGATCTG
*fnb*B	GAACATGGTCAAGCACAAGG	ACGCCATAATTACCGTGACC
*hla*	TCTTGGAACCCGGTATATGG	AGCGAAGTCTGGTGAAAACC
*hlb*	GTGCCAAAGCCGAATCTAAG	ATCAGCGCGTTTATATTGTCC
*hld*	AAGGAAGGAGTGATTTCAATGG	TTTGTTCACTGTGTCGATAATCC
*isa*A	TCCGACAAACACTGTTGACC	AATCCCCAAGCACCTAAACC
*lrg*B	TATTGCCCGAGGATTAGCAC	CAAAGACAGGCACAACTGCTAC
*lyt*M	ACGGTGTCGACTATGCAATG	ATTGCCGCCACCATAGTTAC
*luk*D	GTACTTAAGGCAGCCGGAAAC	CGCCCCAATAAAACTGTGAG
*psm* *α*	TCAAAAGCTTAATCGAACAATTCAC	AATGGCCCCCTTCAAATAAG
*RNAIII*	AAGCCATCCCAACTTAATAACC	GCACTGAGTCCAAGGAAACTAAC
*rpo*B	ACAACCACTTGGCGGTAAAG	ATGCTTCAAGTGCCCATACC
*sar*A	TTGCTTTGAGTTGTTATCAATGG	CAATACAGCGAATTCTTCAAAGC
*sae*R	CCAAGGGAACTCGTTTTACG	ACGCATAGGGACTTCGTGAC
*sig*B	TGATCGCGAACGAGAAATC	ATTGCCGTTCTCTGAAGTCG
